# Epigenetic mechanisms linking atherosclerosis to ischemic stroke: insights from DNA methylation and transcriptome integration

**DOI:** 10.3389/fgene.2025.1567951

**Published:** 2025-06-18

**Authors:** Binrong Ding, Yiqun Wang, Junfeng Li, Xuewei Zhang, Zhengqing Wan, Hao Wang

**Affiliations:** ^1^ People’s Hospital of Ningxiang City, Hunan University of Chinese Medicine, Changsha, China; ^2^ Department of Geriatrics, The Third Xiangya Hospital of Central South University, Changsha, China; ^3^ Health Management Center, Xiangya Hospital, Central South University, Changsha, China; ^4^ National Clinical Research Center for Geriatric Disorders, Xiangya Hospital, Central South University, Changsha, China; ^5^ Center for Medical Genetics and Hunan Key Laboratory of Medical Genetics, School of Life Sciences, Central South University, Changsha, China; ^6^ Department of Medical Genetics, NHC Key Laboratory of Birth Defect for Research and Prevention, Hunan Provincial Maternal and Child Health Care Hospital, Changsha, China; ^7^ MOE Key Lab of Rare Pediatric Diseases, School of Life Sciences, University of South China, Changsha, China

**Keywords:** ischemic stroke, atherosclerosis, integrated bioinformatics approaches, DNA methylation, transcriptomics, epigenetic regulation

## Abstract

**Background:**

Ischemic stroke (IS) is a major cause of mortality and disability, with atherosclerosis (AS) as a primary risk factor. DNA methylation plays a critical role in AS development, but its regulatory mechanisms remain unclear. This study aims to investigate the epigenetic regulatory mechanisms linking AS and IS by integrating DNA methylation and transcriptome data from public databases.

**Methods:**

This study integrated DNA methylation (GSE46394) and transcriptome data (GSE111782 and GSE162955) from public databases to investigate the molecular mechanisms linking AS and IS. Differentially methylated CpG positions (DMPs) and differentially expressed genes (DEGs) were identified (p < 0.05). Subsequent gene annotation and enrichment analyses were performed to uncover potential molecular mechanisms underlying the relationship between AS and IS.

**Results:**

A total of 5,396 consistent DMPs were identified in aortic and carotid atherosclerotic lesions, with enriched pathways such as MAPK signaling and Hippo signaling. Transcriptome analysis revealed 1,147 DEGs in AS plaques and 1,321 DEGs in IS brain tissues, enriched in pathways including neuroactive ligand-receptor interactions, calcium signaling, and vascular smooth muscle contraction. Overlapping analyses identified shared processes like actin filament polymerization, cell migration, and MAPK cascade regulation, as well as pathways such as adrenergic signaling, and apelin signaling.

**Conclusion:**

This study highlights the pivotal role of epigenetic regulation in AS and IS, uncovering key pathways and molecular processes involved in their progression. Future studies should validate these findings in larger cohorts and integrate multi-omics approaches for a comprehensive understanding.

## 1 Introduction

Ischemic stroke (IS) is an acute cerebrovascular disease, accounting for approximately 87% of all acute cerebrovascular cases (Saini et al., 2021; Tu et al., 2023). Studies have shown that there are over 7.6 million new cases of IS worldwide annually (Feigin et al., 2022; Liu et al., 2025), with 1-year mortality rate of up to 6.0% and disability rate of 14.2%, which seriously threatens the quality of life of patients and imposing a significant economic burden on families and society (Shi, 2021; Tu et al., 2021). Atherosclerosis (AS) is one of the primary causes of IS, and preventing and treating AS is an important strategy to reduce the incidence of IS. Although statins and antiplatelet drugs are actively used clinically for primary and secondary prevention of AS, some patients still develop IS (Maranhao and Ca Leite, 2015). Therefore, it is urgent to explore the mechanisms underlying AS and identify new approaches for the prevention and treatment of IS.

In recent years, an increasing number of studies have found that epigenetic modifications, especially DNA methylation, may play an important role in the occurrence and development of AS (Zaina et al., 2014; Aavik et al., 2015; Yamada et al., 2018; Li et al., 2021; Dai et al., 2022). DNA methylation is a dynamic and reversible modification process that can alter genetic expression without changing the DNA sequence. Numerous studies have demonstrated that abnormal DNA methylation plays an important role in AS. Whole-genome bisulfite sequencing of AS plaques and non-plaques in the aorta revealed that the aorta with atherosclerosis exhibited high methylation at many gene loci, with low methylation changes being relatively rare (Zaina et al., 2014; Yamada et al., 2018). Aavik et al. compared the overall DNA methylation levels of femoral artery atherosclerosis samples with normal breast artery samples by HPLC and found that the overall DNA methylation level of the AS group was significantly lower than those in the normal group (Aavik et al., 2015). Li et al. analyzed the whole-genome DNA methylation profiles of carotid artery plaques in patients with clinical symptoms (recent stroke or transient ischemic attack) and those with asymptomatic conditions (no recent stroke). They observed that differentially methylated positions (DMPs) were predominantly hypomethylated in symptomatic plaques, and these hypomethylated genes were associated with various inflammatory processes (Li et al., 2021).

Other studies have suggested that changes in DNA methylation patterns in AS is an adaptive response of the body to inflammation and are dynamic (Aavik et al., 2019). Current results have demonstrated that DNA methylation may play a key role in the occurrence and progression of AS, although its specific mechanism remain unclear. Therefore, this study aims to explore the potential DNA methylation regulatory mechanism in AS by analyzing and comparing transcriptome data and DNA methylation data from atherosclerotic plaques. Additionally, by integrating IS brain tissue transcriptome data, this study seeks to explore the potential role of DNA methylation regulatory mechanisms in AS progression leading to IS.

## 2 Materials and methods

### 2.1 Data collection

DNA methylation data of atherosclerotic plaques, transcriptome data of IS brain tissues, and related clinical information were obtained from Gene Expression Omnibus (GEO, https://www.ncbi.nlm.nih.gov/geo). Specifically, transcriptome data of atherosclerotic plaques were obtained from the GSE111782 dataset, DNA methylation data from the GSE46394 dataset, and transcriptome data of IS brain tissues from the GSE162955 dataset. The GSE111782 dataset includes samples from nine symptomatic patients (disease group) and nine asymptomatic patients (control group) who underwent carotid endarterectomy (CEA). Patients were classified as symptomatic if they suffered a transient ischemic attack, transient monocular blindness ipsilateral to the study artery, or minor or non-disabling ipsilateral stroke. Otherwise, they were classified as asymptomatic. The GSE46394 dataset consists of 34 atherosclerosis samples, including 19 from carotid atherosclerotic samples and 15 from aortic atherosclerotic lesion samples (disease group), along with 15 from aortic tissue samples (control group). The relevant donor (age and sex) and sample information for aortic and carotid specimens are shown in Additional file 1: Supplementary Table S1 in Zaina et al. (2014). The GSE162955 dataset comprises samples from 6 IS patients who died during hospitalization. It includes six tissue samples from infarct sites (disease group) and six tissues from contralateral brain regions (control group). Donor information is shown in the Supplementary Material: Supplementary Table S1.

### 2.2 Differential CpG sites and gene expression analysis

Beta values for CpG probes in the GSE46394 dataset were obtained from the GEO database (https://www.ncbi.nlm.nih.gov/geo/query/acc.cgi?acc=GSE46394). M values, which are log-transformed beta values and more suitable for common statistical tests (Bock, 2012), were used to identify CpG sites with significant methylation differences. Log2 GC-RMA values for GSE111782 (https://www.ncbi.nlm.nih.gov/geo/query/acc.cgi?acc= GSE111782), and RMA mean signal values for GSE162955 (https://www.ncbi.nlm.nih.gov/geo/query/acc.cgi?acc= GSE162955) were obtained from the GEO. Differentially expressed genes (DEGs) were identified using a p-value threshold of α = 0.05. To identify differentially methylated positions (DMPs), we focused on the 367,977 CpG sites annotated to protein-coding genes using HOMER software (Heinz et al., 2010). A liberal genome-wide significance threshold of *p* = 1 × 10^−6^ was applied (Saffari et al., 2018), considering that the study aimed to uncover epigenetic regulatory mechanisms linking AS and IS by integrating different datasets.

### 2.3 Gene annotation and enrichment analysis

Gene annotation was performed using HOMER software (Heinz et al., 2010), which DMPs with nearby genes to facilitate functional annotation. Gene enrichment analysis was conducted using the Gene Ontology (GO) and Kyoto Encyclopedia of Genes and Genomes (KEGG) pathway via Enrichr (https://maayanlab.cloud/Enrichr/) (Chen et al., 2013; Kuleshov et al., 2016; Xie et al., 2021).

### 2.4 Statistical analysis

All statistical analyses were performed using R (version 4.0.3) (Team, 2015). The comparison was conducted using the Wilcoxon rank-sum test from the R package coin (version 1.4.2) (Hothorn et al., 2006). A *p*-value < 0.05 was considered statistically significant for identifying DEGs, while a *p*-value < 1.0 × 10^−6^ was used to determine significantly DMPs.

## 3 Results

### 3.1 AS related DMPs and enrichment analysis

**FIGURE 1:**
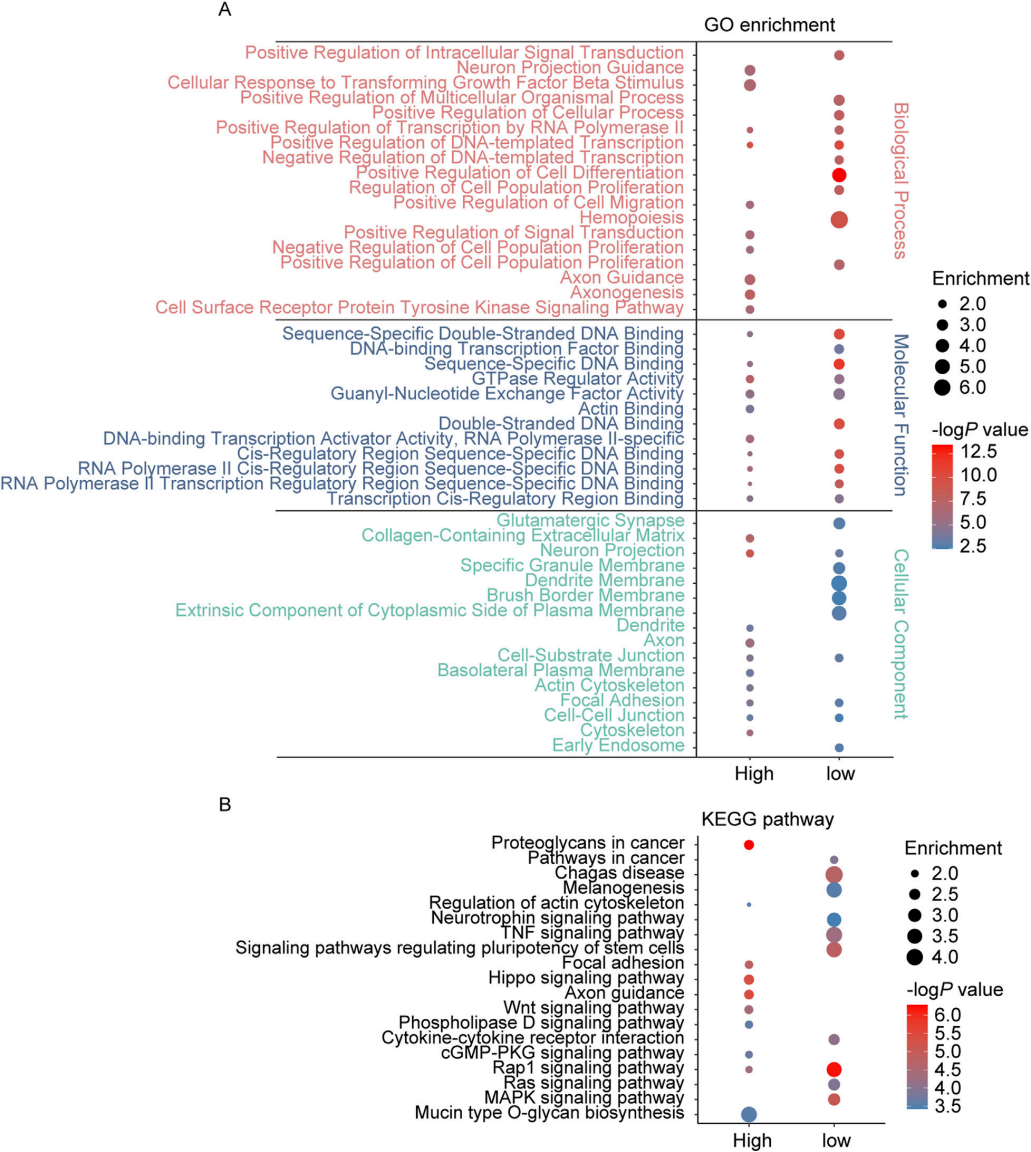
GO and KEGG pathway enrichment analysis of the genes annotated to DMPs in AS. **(A)** The top 10 enriched GO terms of the BP, MF, and CC categories of hypermethylated-DMPs (high) and hypomethylated-DMPs (low); **(B)** The KEGG pathway enriched by hypermethylated-DMPs (high) and hypomethylated-DMPs (low) in AS.

The GSE46394 dataset comprises 19 carotid atherosclerotic samples, 15 aortic atherosclerotic lesion samples, and 15 aortic tissue samples (control group). To identify potential mechanisms in the progression of AS, we conducted differential analysis comparing aortic atherosclerotic lesion samples with the control group and carotid atherosclerotic samples with the control group. AS related DMPs were defined as those showing consistent differences in both comparisons. By *p* < 1.0 × 10^−6^, 5,503 DMPs were identified between aortic atherosclerotic lesion samples and the control samples, while 45,028 DMPs were identified between carotid atherosclerotic samples and the control samples (Supplementary Tables S2a,b). Among the 5,396 overlapping DMPs identified in both datasets, all showed consistent trends in methylation level changes. Of these, 4,415 were hypermethylated related to 2,898 unique protein-coding genes, and 981 were hypomethylated related to 723 unique protein-coding genes (Supplementary Table S2c). Enrichment analysis revealed that the protein-coding genes associated with these DMPs were primarily enriched in biological processes (BP) such as axonogenesis, axon guidance, regulation of signal transduction, tyrosine kinase signaling pathway, cell differentiation, cell migration, cell population proliferation, neuron projection morphogenesis, and transcriptional regulation, as well as molecular functions (MF) including GTPase regulator activity, DNA binding, protein kinase activity, and cadherin binding. The top 10 cellular component (CC) were primarily distributed in axon, cell-cell junction, cell-substrate junction, dendrite, focal adhesions, neuron projection, and synapse. Furthermore, these genes are also linked to KEGG pathways such as axon guidance, cGMP-PKG signaling pathway, focal adhesion, Hippo signaling pathway, MAPK signaling pathway, neurotrophin signaling pathway, phospholipase D signaling pathway, Rap1 signaling pathway, Ras signaling pathway, signaling pathways regulating pluripotency of stem cells, and Wnt signaling pathway (Figure 1).

### 3.2 Atherosclerotic related DEGs and enrichment analysis

**FIGURE 2:**
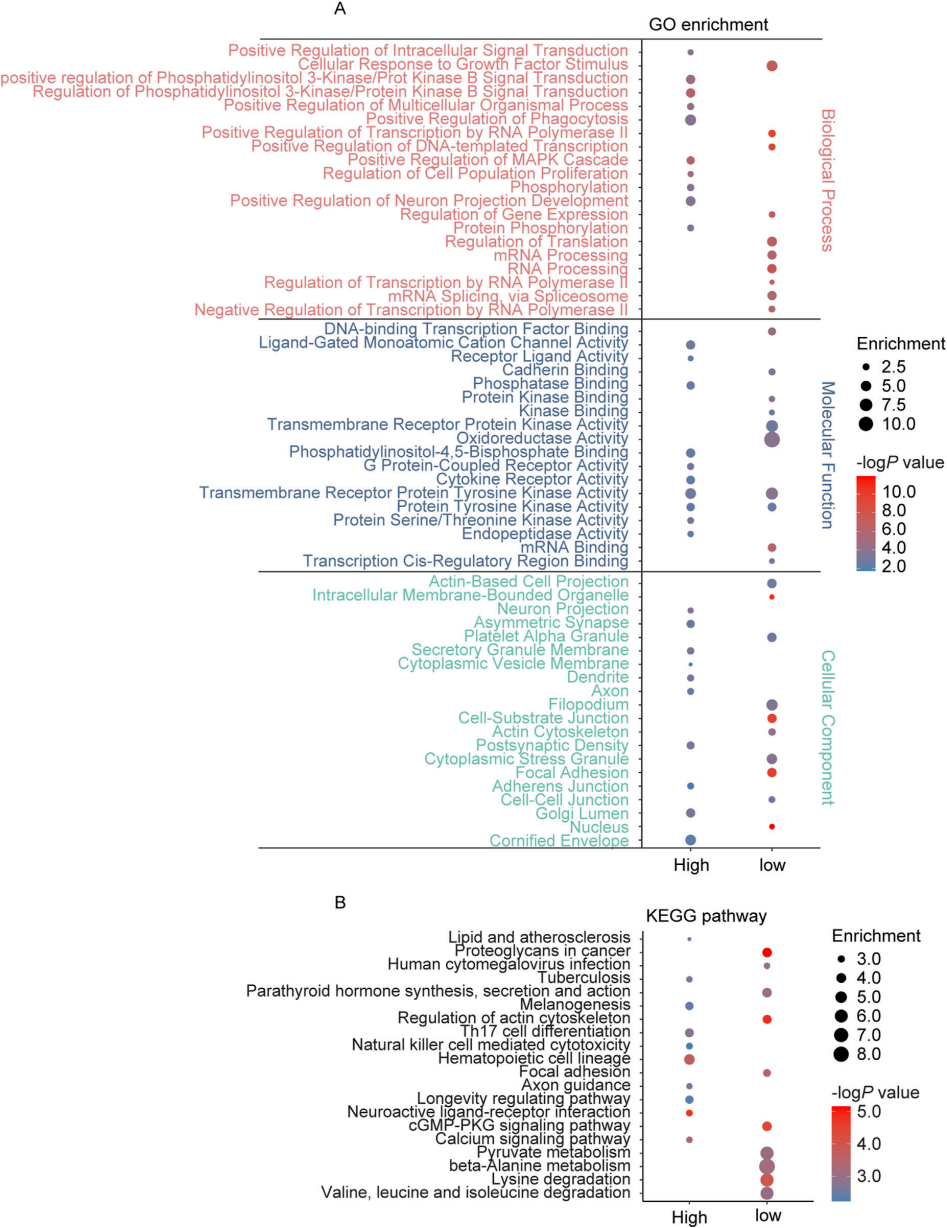
GO and KEGG pathway enrichment analysis of the genes annotated to DEGs in AS. **(A)** The top 10 enriched GO terms of the BP, MF, and CC categories of upregulated DEGs (high) and low-regulated DEGs (low); **(B)** The KEGG pathway enriched by upregulated DEGs (high) and low-regulated DEGs (low) in AS.

The GSE111782 dataset includes samples from 9 IS patients (disease group) and 9 cases with asymptomatic carotid plaques (control group). Using a threshold of 0.05, 1,147 DEGs (967 unique protein code genes) were identified, comprising 597 upregulated genes (503 unique protein-coding genes) and 550 downregulated genes (467 unique protein-coding genes) (Supplementary Table S3). Enrichment analysis revealed that these DEGs were predominantly associated with biological processes such as regulation of multicellular organismal process, regulation of cell population proliferation, tyrosine kinase signaling pathways, regulation of ERK1 and ERK2 cascade, regulation of MAPK cascade, and regulation of neuron projection development. In terms of molecular functions, the DEGs were enriched in protein tyrosine kinase activity, G protein-coupled receptor activity, DNA binding, and cadherin binding. Cellular component analysis indicated that DEGs were linked to cell-cell junction, neuron projections, synapse, and focal adhesions. KEGG pathway analysis further indicated significant enrichment in neuroactive ligand-receptor interaction, axon guidance, calcium signaling pathway, focal adhesions, and longevity regulation pathway (Figure 2).

### 3.3 IS related DEGs and enrichment analysis

**FIGURE 3:**
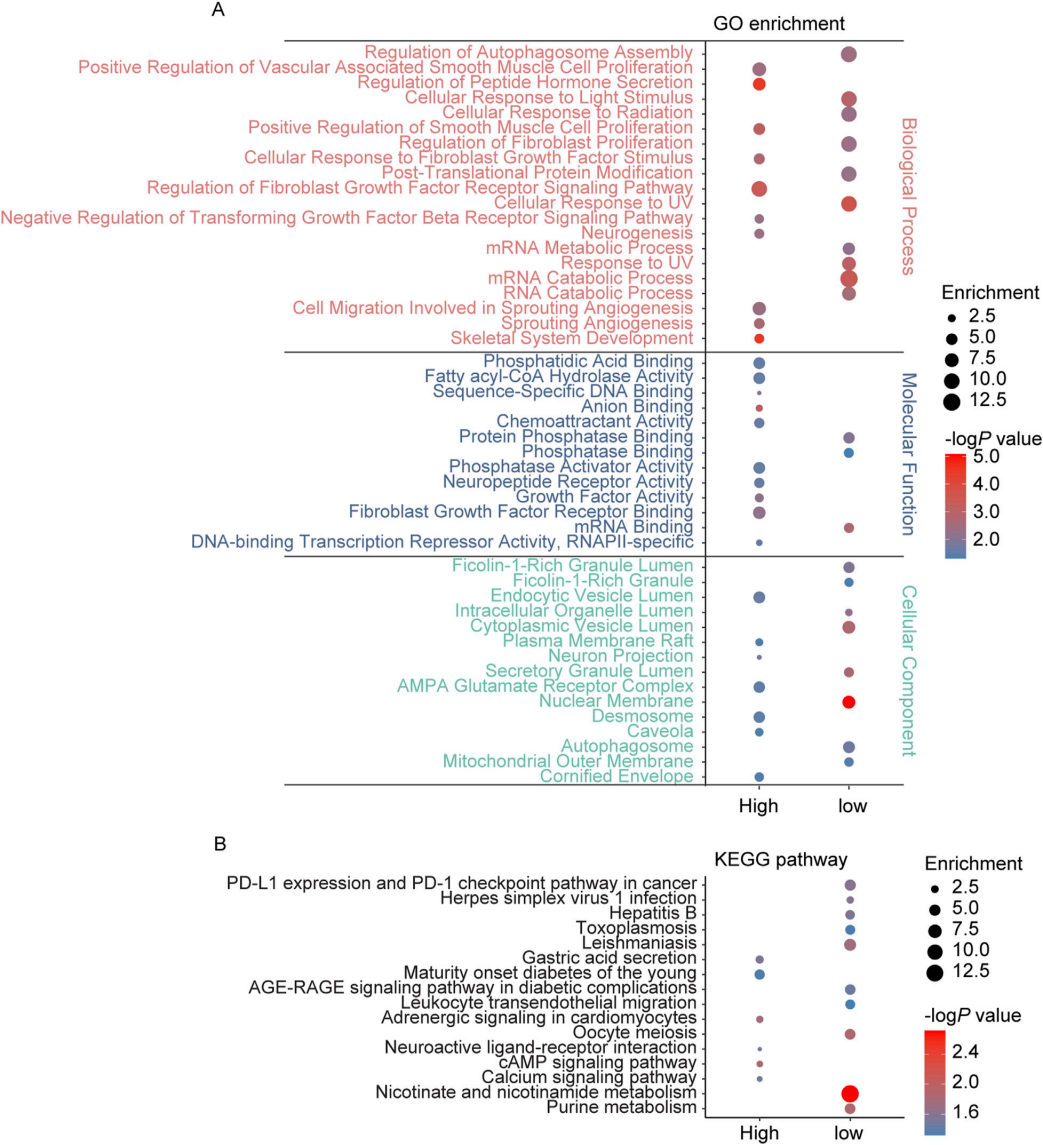
GO and KEGG pathway enrichment analysis of the genes annotated to DEGs in IS. **(A)** The top 10 enriched GO terms of the BP, MF, and CC categories of upregulated DEGs (high) and low-regulated DEGs (low); **(B)** The KEGG pathway enriched by upregulated DEGs (high) and low-regulated DEGs (low) in IS.

The GSE162955 dataset includes brain tissue samples from 6 IS patients, comprising six tissues from contralateral brain regions (disease group) and six tissues from infarct sites (control group). By *p* < 0.05, a total of 1,321 DEGs were identified, including 1,104 upregulated genes (608 unique protein-coding genes) and 217 downregulated genes (142 unique protein-coding genes) (Supplementary Table S4). GO enrichment analysis showed that these DEGs were primarily associated with biological processes such asregulation of autophagosome assembly, regulation of smooth muscle cell proliferation, neurogenesis, sprouting angiogenesis, mRNA metabolic process and post-translational protein modification. In terms of molecular functions, the DEGs were enriched in DNA binding, mRNA binding, phosphatase binding and protein phosphatase regulator activity. For cellular components, DEGs were enriched in neuron projection, autophagosome, vesicles and mitochondria. KEGG pathway analysis indicated significant enrichment in neuroactive ligand-receptor interaction, cAMP signaling pathway, adrenergic signaling in cardiomyocytes, and calcium signaling pathway (Figure 3).

### 3.4 Potential DNA methylation regulatory pathways in AS

**FIGURE 4:**
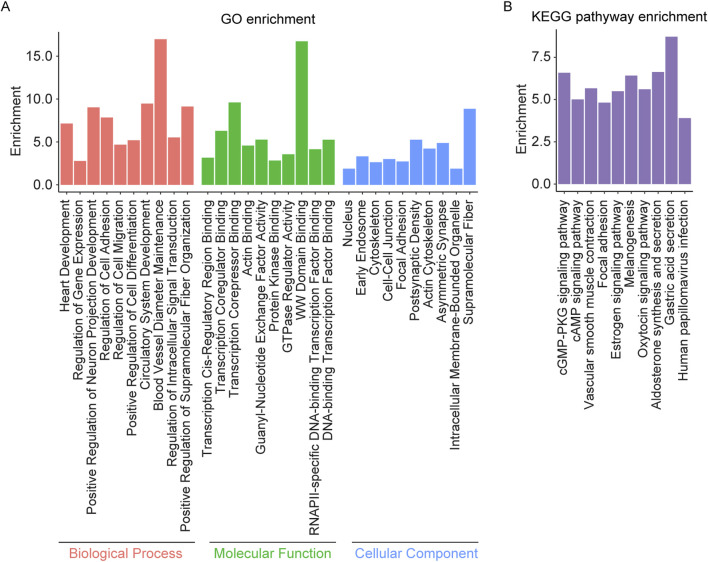
GO and KEGG pathway enrichment analysis of the overlapping genes between AS related DMPs and DEGs. **(A)** The top 10 enriched GO terms of the BP, MF, and CC categories of overlapping genes; **(B)** The KEGG pathway enriched by the overlapping genes.

Enrichment analysis was performed on the overlapping genes between AS related DMPs and DEGs (Supplementary Table S2d). The results indicated significant enrichment in biological processes such as regulation of intracellular signal transduction, regulation of multicellular organismal process, cell differentiation, cell adhesion, cell migration, neuron projection development, and circulatory system development. In terms of molecular functions, the overlapping genes were associated with DNA binding, transcription factor binding, actin binding, and GTPase regulator activity. The cellular components identified included nucleus, cytoskeleton, axon, synapse, dendrite, endosome, neuron projection, cell-cell junction, focal adhesion, and cytoplasmic vesicle membrane. KEGG pathway analysis further revealed enrichment in focal adhesion, cAMP signaling pathway, cGMP-PKG signaling pathway, estrogen signaling pathway, the oxytocin signaling pathway, and vascular smooth muscle contraction (Figure 4).

**FIGURE 5:**
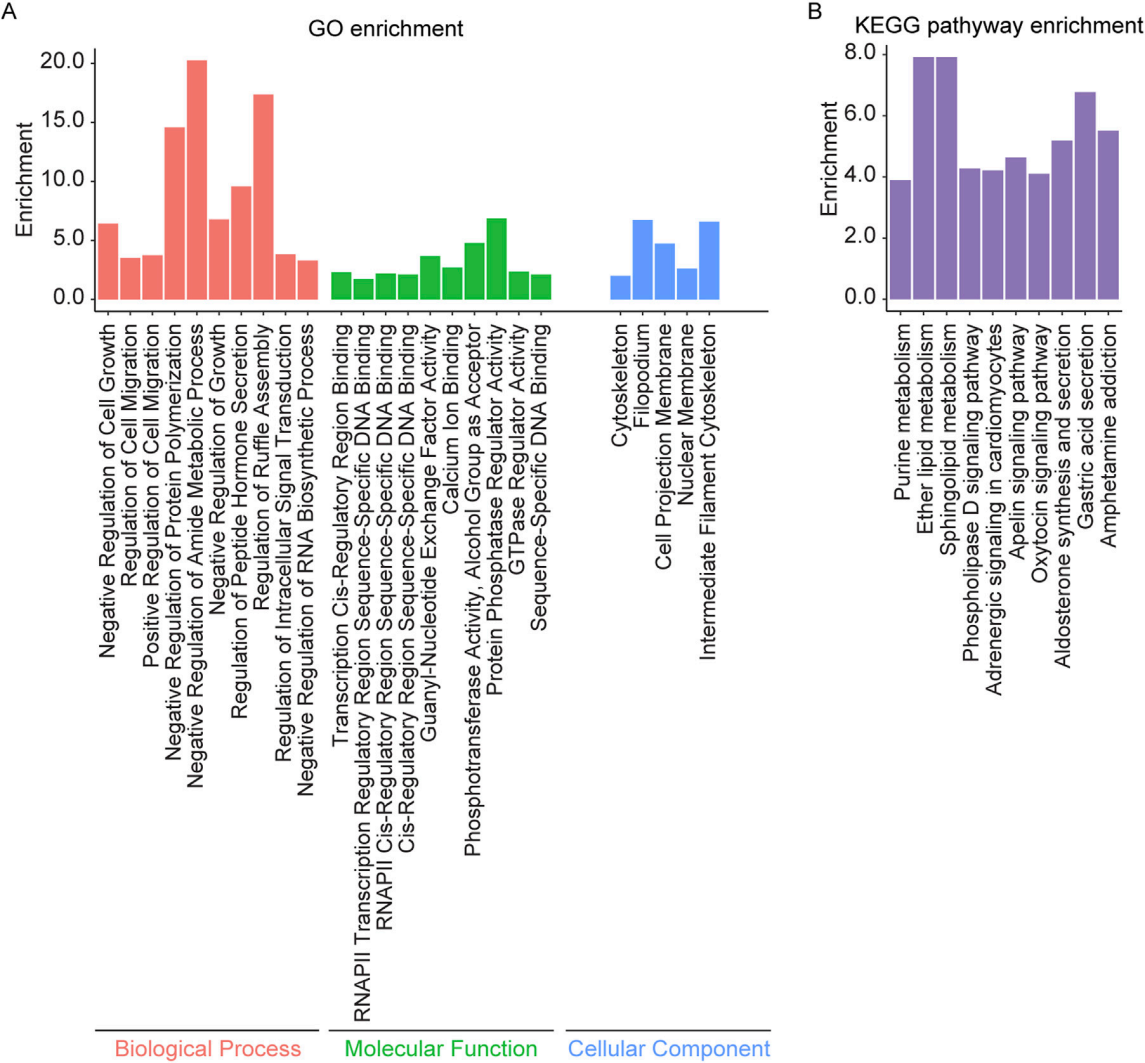
GO and KEGG pathway enrichment analysis of the overlapping genes between AS related DMPs and IS related DEGs. **(A)** The top 10 enriched GO terms of the BP, MF, and CC categories of overlapping genes and **(B)** Top 10 enriched KEGG pathways of overlapping genes.

Enrichment analysis was conducted on the overlapping genes between AS related DMPs and IS related DEGs (Supplementary Table S2e). The results revealed significant enrichment in biological processes such as regulation of cell migration, cell growth, actin filament polymerization, regulation of intracellular signal transduction, and regulation of protein polymerization. Molecular functions included DNA binding, ubiquitin protein ligase activity, transcription factor binding, calcium ion binding, protein phosphatase regulator activity, and GTPase regulator activity. Enrichment was observed in cellular components such as the intermediate filament cytoskeleton, cell projection membrane, cytoskeleton, nuclear membrane, and filopodium. KEGG pathway analysis showed enrichment in the apelin signaling pathway, sphingolipid metabolism, phospholipase D signaling pathway, adrenergic signaling in cardiomyocytes, aldosterone synthesis and secretion, oxytocin signaling pathway, and purine metabolism (Figure 5).

**FIGURE 6:**
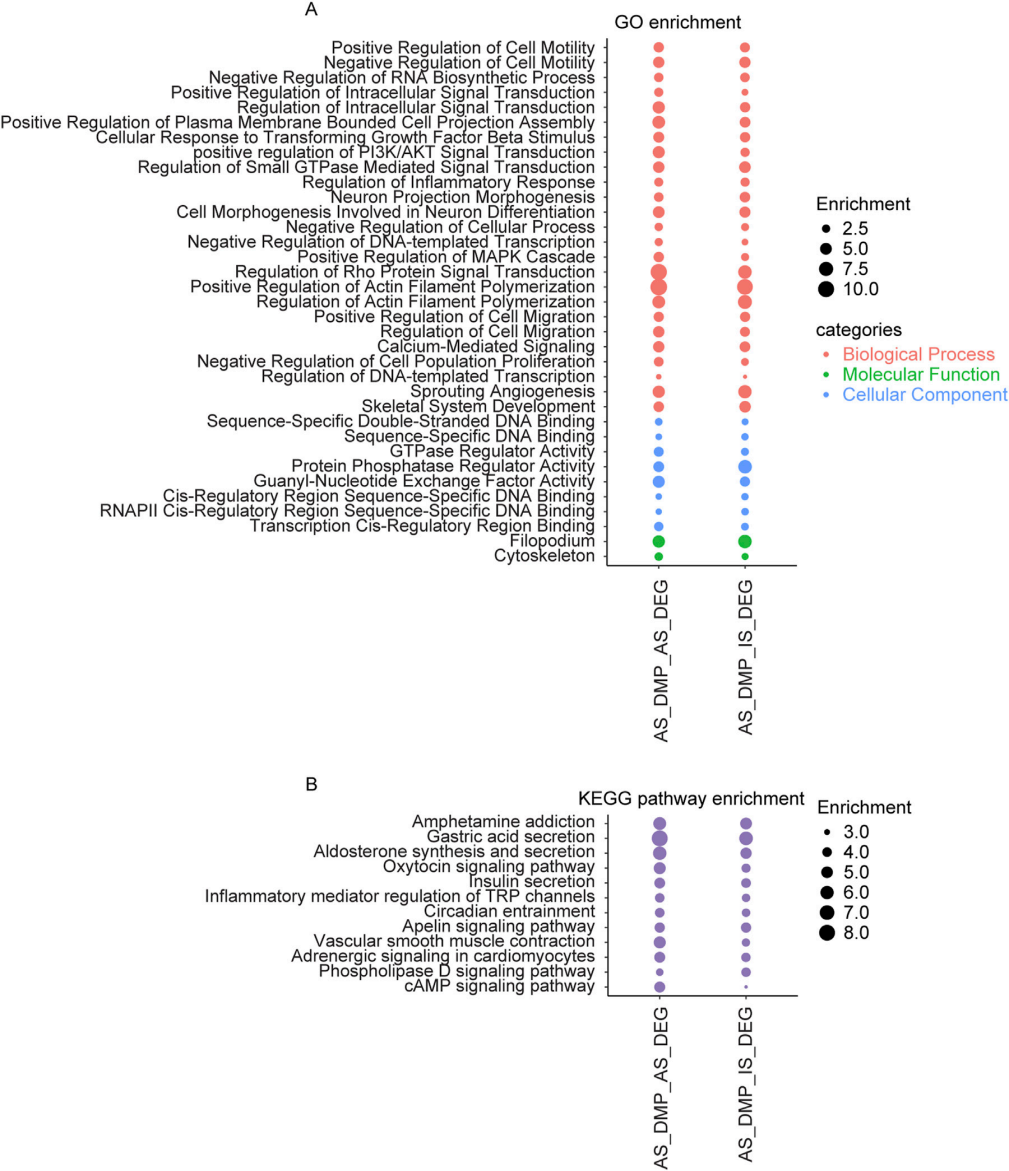
The overlapping items in the enrichment results of genes shared between AS related DMPs and DEGs, and between AS related DMPs and IS related DEGs. **(A)** Overlapping results in GO terms of the BP, MF, and CC categories and **(B)** Overlapping results in KEGG pathway. AS_DMP_AS_DEG: enrichment results of genes shared between AS related DMPs and DEGs; AS_DMP_IS_DEG: enrichment results of genes shared between AS related DMPs and IS related DEGs.

A comparison of enrichment results for overlapping genes between AS related DMPs and DEGs versus those between AS related DMPs and IS related DEGs revealed several shared enriched categories. Commonly enriched biological processes include regulation of cell migration, regulation of actin filament polymerization, regulation of cell motility, regulation of inflammatory response, and regulation of the MAPK cascade, regulation of PI3K/AKT signal transduction, Neuron Projection Morphogenesis. Shared molecular functions include DNA binding and GTPase regulator activity. Enrichment was observed in cellular components such as the cytoskeleton and filopodium. KEGG pathway commonly enriched include adrenergic signaling in cardiomyocytes, aldosterone synthesis and secretion, apelin signaling pathway, cAMP signaling pathway, circadian entrainment, insulin secretion, oxytocin signaling pathway, phospholipase D signaling pathway, and vascular smooth muscle contraction (Figure 6).

## 4 Discussion

This study provides a comprehensive analysis of the molecular mechanisms underlying AS and IS by integrating DNA methylation and gene expression data. It highlights the critical role of epigenetic regulation in both diseases and uncovers key biological pathways and processes involved in their progression.

The identification of 5,396 DMPs with consistent trends in methylation level changes highlights the significant epigenetic changes underlying the progression of AS across different vascular regions. These DMPs are associated with genes involved in axon guidance, cell migration, and signal transduction, which are essential processes in vascular remodeling and endothelial dysfunction (Munoz-Chapuli et al., 2004; Eichmann et al., 2005; Michaelis, 2014). The enrichment of pathways such as Hippo signaling, Wnt signaling, MAPK signaling, Ras signaling and Rap1 signaling suggests that epigenetic modifications may drive the progression of AS by altering cellular behaviors such as migration and adhesion, which are crucial for maintaining vascular integrity and responding to mechanical stress. Notably, the Wnt, MAPK and Hippo signaling pathways are known to regulate key processes such as cell proliferation, survival, and migration (Chien et al., 2009; Fu et al., 2022; Bahar et al., 2023). Abnormal activation of the Wnt signaling pathway has been shown to promote vascular remodeling and plaque formation (Poznyak et al., 2023; Khan et al., 2024; Kocełak et al., 2024). Similarly, the MAPK signaling pathway is activated in response to inflammatory factors (e.g., TNF-α, IL-6) or oxidative stress, leading to abnormal proliferation or apoptosis of vascular smooth muscle cells (VSMCs) and endothelial cells, and is closely related to the formation and instability of atherosclerotic plaques (Wu et al., 2018). Dysregulation of the Hippo signaling pathway may lead to abnormal proliferation or excessive apoptosis of vascular wall cells, thereby destroying the integrity of vascular structure and promoting the occurrence of lesions. Additionally, the enrichment of focal adhesion and cytoskeletal regulation pathways suggests a significant role in maintaining vascular integrity. Focal adhesion kinase (FAK), a key component of the focal adhesion complex, could promotes NF-κB–driven inflammation in atherosclerosis (Murphy et al., 2023). These processes are essential for endothelial cell function and the ability of VSMCs to migrate and proliferate in response to injury. DNA methylation is related to different stages of AS. In the early stages of AS, DNA methylation may primarily regulate inflammatory responses and endothelial function, whereas in later stages, it may be associated with macrophages, foam cell formation, VSMCs proliferation, and ultimately, plaque rupture and thrombosis (Zhang et al., 2021). In this study, we found that these pathways likely play critical roles in endothelial dysfunction and vascular remodeling, further supporting their importance as potential epigenetic regulatory targets in the pathology of AS.

The study explores the dysregulated cellular processes in both AS and IS, identifying common pathways such as regulation of cell migration, actin filament polymerization, positive regulation of cell motility, regulation of inflammatory response, regulation of PI3K/AKT signal transduction, ubiquitin protein ligase activity, and MAPK cascade regulation. These findings underscore the importance of cellular dynamics and inflammation in the progression of both diseases. Cell migration and motility are essential for endothelial repair and smooth muscle cell proliferation, of which contribute to vascular remodeling and lesion formation (Fonseca et al., 2020; Liu and Lin, 2022). Chronic inflammation and dysregulated MAPK signaling are central to the initiation and progression of AS and IS, influencing vascular integrity and immune responses (Wu et al., 2018; Kong et al., 2022; Totoń-Żurańska et al., 2024). The PI3K/AKT pathway is involved in multiple biological processes, including endothelial cell apoptosis, lipid accumulation and transport, macrophage autophagy, phenotypic transition, and excessive smooth muscle proliferation (Zhao et al., 2021). Ubiquitin protein ligase activity is important in the ubiquitination process. Ubiquitination contributes to AS pathogenesis through the regulation of vascular inflammation, endothelial cell and vascular smooth muscle cell function, lipid metabolism and atherosclerotic plaque stability (Zhou et al., 2021). The common enrichment of DNA binding indicates the importance of gene regulation, likely influenced by epigenetic modifications such as DNA methylation, in modulating cellular behavior. The common enrichment of cytoskeleton and filopodia further emphasizes their critical roles in endothelial barrier function, mechanotransduction, and the migration of vascular and immune cells, all of which are vital during plaque formation and ischemic injury. Additionally, commonly enriched pathways, such as neuroactive ligand-receptor interactions, adrenergic signaling, and calcium signaling, suggest overlapping regulatory mechanisms related to vascular and neural functions in both AS and IS. The dysregulation of tyrosine kinase signaling pathways in both diseases may contribute to abnormal cell proliferation and migration, processes crucial for the pathogenesis of vascular diseases. In particular, VSMCs and ECs are key players in vascular remodeling, and their dysfunction can exacerbate the progression of AS and IS (Méndez-Barbero et al., 2021). Adrenergic signaling plays a pivotal role in regulating cardiovascular responses to stress (Wong et al., 2008; Ali et al., 2020), but its overactivation may result in vascular remodeling, oxidative stress, and inflammatory responses, all of which are central to the progression of AS and IS. The involvement of calcium signaling in both diseases is noteworthy. Dysregulated calcium homeostasis can lead to altered vascular tone and endothelial dysfunction, exacerbating oxidative stress and inflammation (Pacinella et al., 2022). These changes are key contributors to both AS and IS, suggesting that targeting calcium signaling could be a potential therapeutic avenue.

While this study highlights several overlapping pathways between AS and IS, it also underscores the distinct features between the two diseases. For instance, the study mentions mitochondrial processes that are enriched in IS related DEGs, which likely reflect tissue-specific responses to ischemia. Both diseases show common involvement in pathways such as MAPK cascade activation, actin filament assembly, and inflammatory responses which are crucial for the adaptive response of vascular cells to injury and stress (Wu et al., 2020; Ravarotto et al., 2022; Espina et al., 2023), and their dysregulation in both AS and IS highlights the systemic impact of these diseases on vascular and neural functions. The identification of mitochondrial processes in IS related DEGs suggests a more complex, tissue-specific response to ischemia. This finding implies that IS may involve unique molecular mechanisms related to energy metabolism and cell survival under ischemic conditions, which are distinct from those in AS.

The integration of DNA methylation and gene expression data highlights several potential therapeutic targets, including apelin signaling and sphingolipid metabolism. These pathways are involved in vascular inflammation and endothelial function, making them promising targets for therapeutic intervention in both AS and IS. Apelin is involved in regulating vascular tone, endothelial function, and angiogenesis (Chapman et al., 2023). Given its role in vascular diseases, targeting apelin signaling could help modulate vascular inflammation and endothelial dysfunction in AS and IS. Similarly, sphingolipids are critical in regulating cellular processes like proliferation, migration, and apoptosis (Iessi et al., 2020). Their involvement in vascular inflammation and endothelial dysfunction makes them a potential target for interventions aimed at controlling the progression of AS and IS.

## 5 Limitations

While this study provides valuable insights into the epigenetic regulation of AS and IS, it has several limitations. First, this study focuses solely on these two disease states without considering the characteristics of DNA methylation at different pathological stages of AS and IS. Since DNA methylation patterns vary across different tissue types and disease states, these variations may influence disease onset and progression. Additionally, this study primarily examined shared epigenetic modifications across different vascular regions, without accounting for region-specific DNA methylation characteristics. Given these differences, future research should further refine the analysis of DNA methylation characteristics across distinct pathological stages and vascular regions to provide a more nuanced understanding of how epigenetic modifications contribute to the onset and progression of AS and IS. Second, as this study relied on publicly available datasets, potential heterogeneity in sample collection, patient demographics, and clinical conditions may introduce bias. Future studies should account for these potential confounders. Additionally, while we identified pathways associated with DNA methylation, functional validation is necessary to confirm their role in AS and IS. Finally, this study focused on DNA methylation and transcriptomic alterations but did not integrate other omics data, such as proteomics or metabolomics, which could provide a more comprehensive understanding of plaque instability. Future research should adopt a multi-omics approach to explore the interplay between genetic, epigenetic, and metabolic factors in the pathogenesis of AS and IS.

## 6 Conclusion

In conclusion, this study provides insights into the epigenetic mechanisms underlying AS and their potential role in IS. The integration of methylation and gene expression data highlights key pathways and processes that may serve as targets for prevention and treatment strategies. These findings emphasize the importance of addressing both local vascular changes and systemic factors in effective managing AS and IS.

## Data Availability

The original contributions presented in the study are included in the article/Supplementary Material, further inquiries can be directed to the corresponding authors.
